# Special Issue in “Nanomaterials and Their Applications in Sensing and Biosensing”

**DOI:** 10.3390/bios13060625

**Published:** 2023-06-06

**Authors:** Shabi Abbas Zaidi, Faisal Shahzad, Asad Abbas

**Affiliations:** 1 Department of Chemistry and Earth Sciences, College of Arts and Sciences, Qatar University, Doha 2713, Qatar; 2 Department of Metallurgy & Materials Engineering (DMME) PIEAS, PO Nilore, Islamabad 44000, Pakistan; 3 Institute for Sustainable Energy and the Environment, Department of Chemical and Biomolecular Engineering, Ohio University, Athens, OH 45701, USA

The identification of the target molecule is required for rapid and reliable clinical diagnosis and disease monitoring [1]. Biosensing has emerged as a versatile technique for the early detection of health-related issues. The biosensing technique is not only rapid and reliable, but it also offers high sensitivity and can be used to investigate a variety of analytes [2]. Nanomaterials have been widely exploited in the preparation of sensors and biosensors owing to their excellent features, such as high surface area, quantum size effect, and active surface [3]. This Special Issue focused on the different designs of nanomaterials and their roles in constructing sensing and biosensing platforms for detecting various analytes. 

Surface plasmon resonance (SPR) has emerged as one of the prevalent label-free biosensing technologies. Zakirov et al. [4] designed nanomaterials hybrid structures based on copper, graphene, and two-dimensional transition metal dichalcogenides (TMDC) as an ultrasensitive biosensing platform. Theoretical and numerical calculations were performed using the Goos–Hänchen (GH) shift method. The GH shift is the lateral position displacement of the p-polarized reflected beam from a boundary of two media having different refractive indices under total internal reflection conditions. The extent of the lateral position can be found from the phase change of the beam. In this work, authors optimized several parameters, such as the thickness of copper, the thickness of 2D layered materials, and excitation wavelength, and showed an enhanced sensitivity with a detection limit of 10^−9^ refractive index units (RIU). In another report by Zhu et al. [5] the authors proposed an SPR fiber probe to work intravenously, which offers real-time detection of Circulating Tumor Cells (CTCs) in bloodstreams. The SPR probe could detect Michigan Cancer Foundation-7 (MCF-7) breast cancer cells in flowing whole mouse blood with a detection limit of ~1.4 cells per microliter by using an epithelial cell adhesion molecule (EpCAM) antibody-based receptor layer and a 15 min enrichment period.

To overcome the difficulty of detecting smaller biomolecules (less than 400 Da) with conventional SPR sensing techniques, Hedhly et al. [6] developed a highly sensitive SPR biosensor based on the symmetric metal cladding plasmonic waveguide (SMCW) structure. The configuration and thickness of the guiding layer were precisely controlled such that ultra-high order modes can be excited, which generates a steep phase change and a large position shift from the Goos–Hänchen effect. The experimental results depict a lateral position signal change >90 µm for glycerol with a sensitivity FOM of 2.33 × 10^4^ µm/RIU and more than 15 µm for 10^−4^ M biotin. In another submission, Youssef et al. [7] demonstrated the ability of a plasmonic metasensor to detect ultra-low refractive index changes by employing vanadium dioxide (VO_2_) as the sensing layer. The approach was different from the current cumbersome plasmonic biosensing setups based on optical-phase-singularity measurement, as the phase signal detection in this study was based on the direct measurement of the phase-related lateral position shift (Goos–Hänchen) at the sensing interface. The high sensitivity (1.393 × 10^8^ μm/RIU for ∆n = 10^−10^ RIU), based on the Goos–Hänchen lateral shift of the reflected wave, becomes significant when the sensor is excited at resonance due to the near-zero reflectivity dip, which corresponds to the absolute dark point (lower than 10^−6^). As a result, GH shifts in the order of 2.997 × 10^3^ μm were obtained using the optimal metasurface configuration. 

Shanbhag and co-workers [8] developed a biosensor based on graphitic carbon nitride (gCN) for the detection of Pramipexole (PMXL), which is used in treating Parkinson’s disease. However, an increased level of PMXL could lead to severe complications. The gCN-modified carbon paste electrode (gCN/CPE) exhibited a good linearity range from 0.05 to 500 µM, and a lower detection limit (LD) of 0.012 µM. The selectivity of the electrode in PMXL detection was investigated by conducting an interference study, while the tablet sample analysis demonstrates the sensitive and real-time application of the electrode. In a paper submitted by Alrashidi et al. [9] an electrochemical sensor based on palladium nanoparticles deposited in a porous silicon-polypyrrole-carbon black nanocomposite (Pd@PSi-PPy-C)-fabricated over a glassy carbon electrode (GCE) was developed to detect hydroquinone (HQ). The authors achieved excellent sensitivity of 3.0156 μA μM^−1^ cm^−2^ and a low limit of detection (LOD) of 0.074 μM, thus leading to detect wide-ranging HQ (1–450 μM) in neutral pH media. In another similar study, Suaifan et al. [10] developed GCE modified with platinum-palladium nanoparticles for the detection of albendazole (ABZ) in pharmaceutical dosage form and in contaminated animal-derived products. The modified electrode exhibited high sensitivity and applicability, with a lower limit of detection of 0.08 µmol L^−1^ in aqueous solution and 10 µmol L^−1^ in the contaminated ground chicken, and 100 µmol L^−1^ in the contaminated milk sample. Sadrabadi and co-workers [11] developed an electrochemical nanobiosensor to detect alprazolam, averting the undesirable consequences of overdose. GCE was immobilized with Gold nanourchins (AuNUs), and iron-nickel reduced graphene oxide (Fe-Ni@rGO). The sensing platform demonstrated two linear ranges (4 to 500 µg L^−1^ and 1 to 50 mg L^−1^), low limit of detection (1 µg L^−1^), high sensitivity, good repeatability, and good recovery owing to the increased functionalities, including –OH and carboxyl (-COOH) groups, on the electrode surface. 

Faisal et al. [12] developed Pt nanoparticles embedded polypyrrole-carbon black (PPy-CB)/ZnO nanocomposites (NCs) for selective detection of 4-nitrophenol (4-NP). The GCE was modified by [poly(3,4-ethylenedioxythiophene) polystyrene sulfonate; PEDOT:PSS] based on Pt NPs/PPy-CB/ZnO NCs and functioned as the working electrode. The modified electrode exhibited selectivity toward 4-NP and showed linearity from 1.5 to 40.5 µM and LOD of 1.25 ± 0.06 µM. 

In the work submitted by Lee et al. [13] a horseradish peroxidase-encapsulated fluorescent bio-nanoparticle (HEFBNP) comprising bovine serum albumin-stabilized gold nanoclusters (BSA-AuNCs) and horseradish peroxidase-stabilized gold nanoclusters (HRP-AuNCs) were developed for the detection of H_2_O_2_. The HEFBNP could serve two purposes, first is that HEFBNPs have a continuous two-step fluorescence quenching mechanism, and second, the proximity of two protein-AuNCs in a single HEFBNP allows a reaction intermediate (•OH) to rapidly reach the adjacent protein-AuNCs. As a result, the HEFBNP-based sensing system can measure very low concentrations of H_2_O_2_ up to 0.5 nM and show good selectivity. Yu and colleagues [14] developed low-cost and scalable preparation of magnetic nanoparticles (MNPs) from biowaste and demonstrated their successful application in viral RNA extraction and the detection of COVID-19. 

This Special Issue also features a few state-of-the-art reviews. For example, Xu et al. [15] and Rayegani et al. [16] presented the progress in biomolecular detection based on aptamers and nanoparticles and wearable sensors using piezoelectric and triboelectric nanogenerators, respectively. Barani and co-workers [17] submitted an excellent review on the role of nanotechnology in the management of Klebsiella pneumonia-related Infections. In another review, Zaidi and co-workers [18] discussed the excellent limit of detection achieved by molecularly imprinted polymer-based electrochemical sensors. 

In conclusion, different and interesting approaches employing nanomaterials were published in this Special Issue. The findings reported in this Special Issue suggest that nanomaterials possess the potential to be used for the development of various electrochemical sensors and biosensors. We trust that this Special Issue will be useful for the wide scientific community.
